# Letter from the Editor in Chief

**DOI:** 10.19102/icrm.2022.130206

**Published:** 2022-02-15

**Authors:** Moussa Mansour


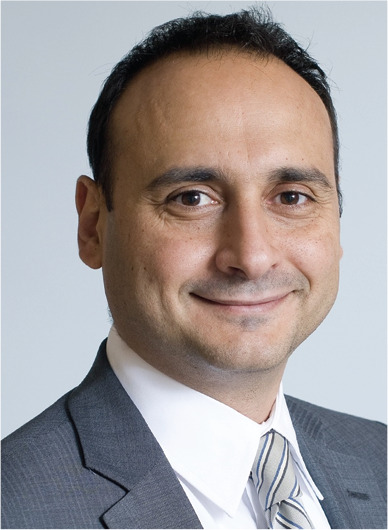


Dear readers,

The use of ambulatory cardiac monitoring has rapidly expanded in the past few years. This issue of *The Journal of Innovations in Cardiac Rhythm Management* contains an interesting article titled “Use of Ambulatory Patch Monitoring Devices to Supplement Inpatient Telemetry—A Descriptive Study of a Single-center Experience During the Coronavirus Disease 2019 Pandemic.” In it, Vanchiere et al.^1^ describe the use of mobile cardiac outpatient telemetry (MCOT) instead of conventional telemetry monitoring for lower-risk non–critically ill patients who are COVID-19–positive. The reasons for cardiac monitoring included, among others, troponin elevation, the use of QT-prolonging medications, and the presence of coronary artery disease or congestive heart failure. The monitoring strategy showed success in detecting a variety of arrhythmias, and the average time between arrhythmia occurrence and notification of the care team was 4 hours and 7 minutes, ranging between 16 minutes and 22 hours.

Despite the long time to notification, this study is important because it highlights a new practice of using ambulatory cardiac monitors instead of conventional telemetry units for low-risk patients. This practice has the potential to decompress telemetry beds, which are fully saturated in many parts of the country because of the COVID-19 pandemic. The long notification time could be shortened by providing additional technical support and designing algorithms to speed up the message delivery, both of which are likely to be less costly than telemetry unit admission. Moreover, MCOT monitors could be used not only to shift patients from telemetry units to regular beds but also to avoid hospital admissions altogether in some cases. It may also be possible to use advanced consumer devices, such as smartwatches, instead of MCOT devices to monitor some groups of patients that are conventionally admitted for low-risk situations. Consumer devices allow patients to directly upload the recorded information to their electronic medical records and send notifications to their health care providers. Artificial intelligence algorithms are also being developed to improve the accuracy of consumer devices and reduce the occurrence of false-positive findings that can place an unnecessary burden on providers.

The widespread use of ambulatory cardiac monitoring, consumer devices, and artificial intelligence will likely transform cardiac health delivery in a major way; among other outcomes, it may reduce the use of telemetry beds and number of hospital admissions and improve patient satisfaction. Clinical studies, such as the one by Vanchiere et al.,^1^ will help provide the necessary guidance in this rapidly moving field.

I hope that you enjoy reading the articles in this issue of the journal.

Sincerely,


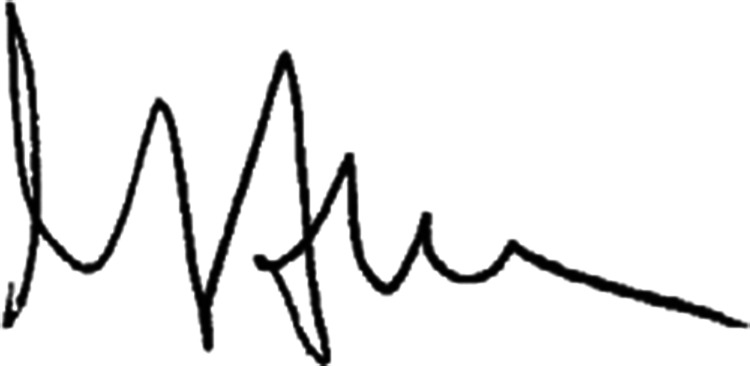


Moussa Mansour, md, fhrs, facc

Editor in Chief

*The Journal of Innovations in Cardiac Rhythm Management*

MMansour@InnovationsInCRM.com

Director, Atrial Fibrillation Program

Jeremy Ruskin and Dan Starks Endowed Chair in Cardiology

Massachusetts General Hospital

Boston, MA 02114
